# Supplementary material for: Morphometric relationships and their contribution to biomass and cannabinoid yield in hybrids of hemp (Cannabis sativa)
Source: J Exp Bot. 2021 Jul 21;72(22):7694–709. doi: 10.1093/jxb/erab346 (PMC8643699; doi:10.1093/jxb/erab346)
Supplement: erab346_suppl_Supplementary_Figures_S1-S8 [file erab346_suppl_supplementary_figures_s1-s8.pdf]

Supplementary Figures

Fig. S1. Genetic diversity. Neighbor-joining tree **(A)** of 190 hemp cultivars, crosses, and U.S. feral accessions, **(B)** allelic richness of DAPC clusters by chromosome (loess span = 0.2), **(C)** genome-wide mean allelic richness, and **(D)** dendrogram of hemp kinship (hclust). Clusters are colored according the legend in panel **(A)**.

Fig. S2. Field design.

Fig. S3. Diagram of architectural traits within 2-D kite. Trait descriptions can be found in Table 2 and Supplementary Table S1.

Fig. S4. Example of masking **(A)** original scanned leaf images using **(B)** GLI <0.5, then (C) calculating mean GLI on the image for the unmasked leaf. Note: Darker regions in **(B)** reflect signs of powdery mildew (*Golovinomyces spadiceus*).

Fig. S5. Data processing pipeline for the extraction of phenotypic traits from RGB and multispectral data. Derived vegetation indices include normalized difference vegetation index (NDVI), enhanced vegetation index (EVI), green chlorophyll index (GCI), green normalized difference vegetation index (GNDVI), modified non-linear index (MNLI), modified soil adjusted vegetation index 2 (MSAVI2), and optimized soil adjusted vegetation index (OSAVI).

Fig. S6. Pairwise correlations of field collected traits with aerial morphological indices on a plot-level (upper triangle) and family-level (lower triangle) basis. The color of coefficients within cells represent significant ($p < 0.01$) positive (blue) or inverse (red) correlations.

Fig. S7. Pairwise correlations of field collected traits. The color of each square in **(A)** represents a significant ($p < 0.01$) positive (blue) or inverse (red) correlation. The size of each square represents the strength of the correlation. Non-significant correlations ($p < 0.01$) were not drawn. Traits were ordered via hierarchical clustering (method = “complete”). PCA biplot **(B)** of the same traits (scaled) using family means. Descriptions of trait abbreviations are listed in Table 2 and Table S1. Cannabinoid data are not shown.

Fig. S8. Pairwise correlations of cannabinoid profiles and aerial indices over time. The color of each square in **(A)** represents a significant ($p < 0.01$) positive (blue) or inverse (red) correlation. The size of each square represents the strength of the correlation. Non-significant correlations ($p < 0.01$) were not drawn.

Fig. S1.

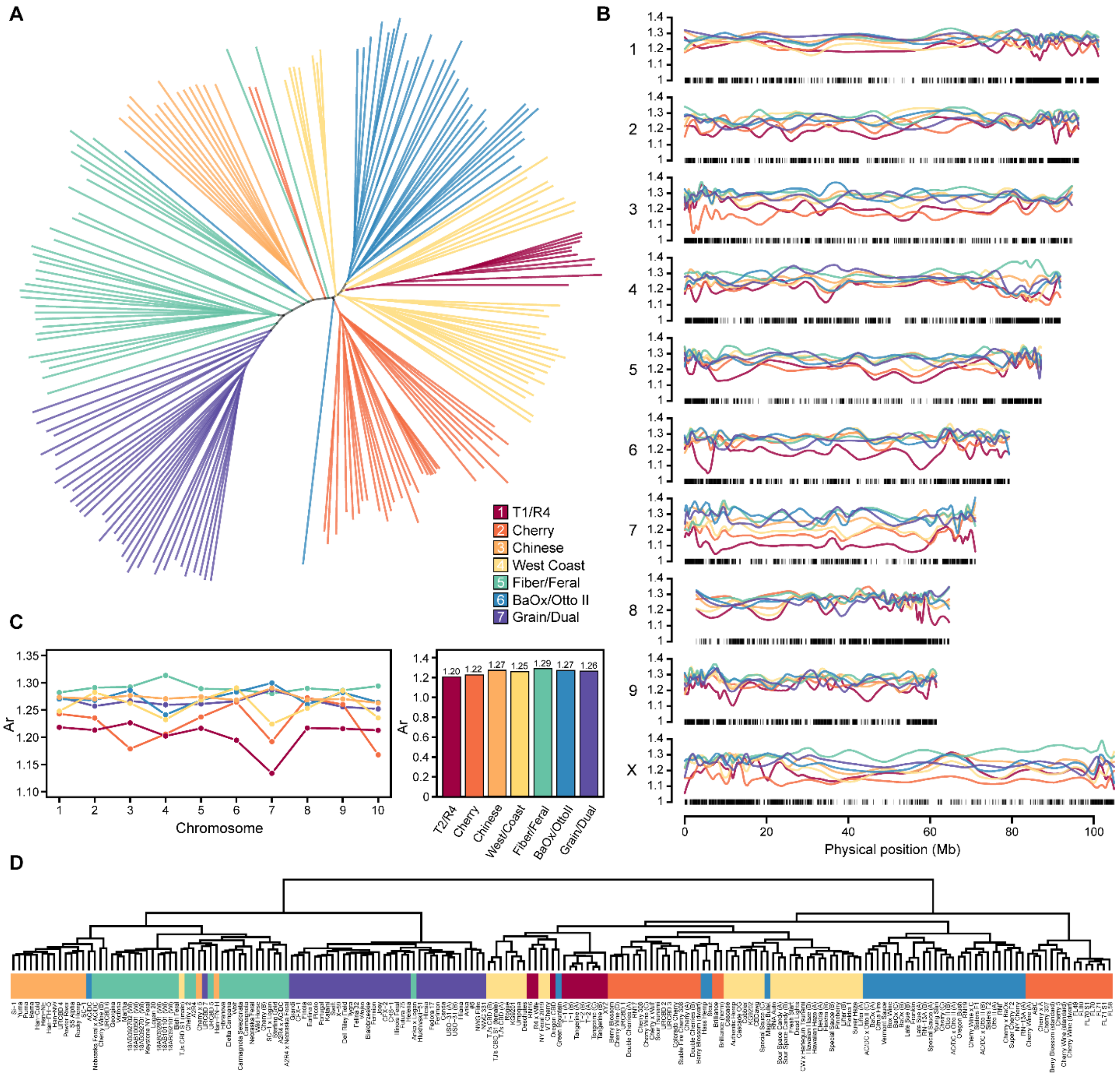
A
B
C
D
1 T1/R4
2 Cherry
3 Chinese
4 West Coast
5 Fiber/Feral
6 BaOx/Otto II
7 Grain/Dual
Ar
Chromosome
Physical position (Mb)
1.20
1.22
1.27
1.25
1.29
1.27
1.26
T2/R4
Cherry
Chinese
West/Coast
Fiber/Feral
BaOx/OttoII
Grain/Dual

Fig. S2.

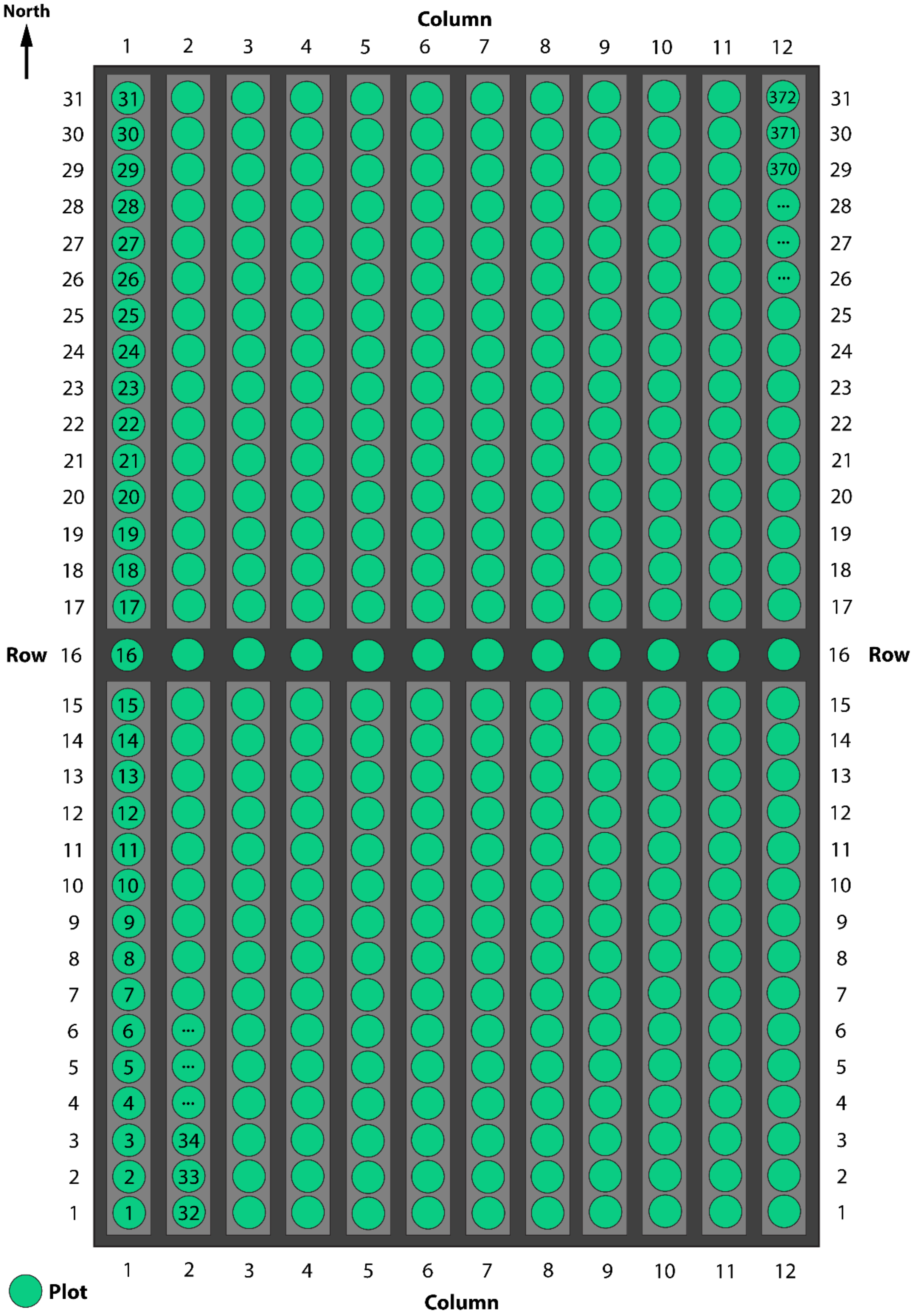
North
Column
1 2 3 4 5 6 7 8 9 10 11 12
Row
31 30 29 28 27 26 25 24 23 22 21 20 19 18 17 16 15 14 13 12 11 10 9 8 7 6 5 4 3 2 1
372
371
370
34
33
32
Plot

Fig. S3.

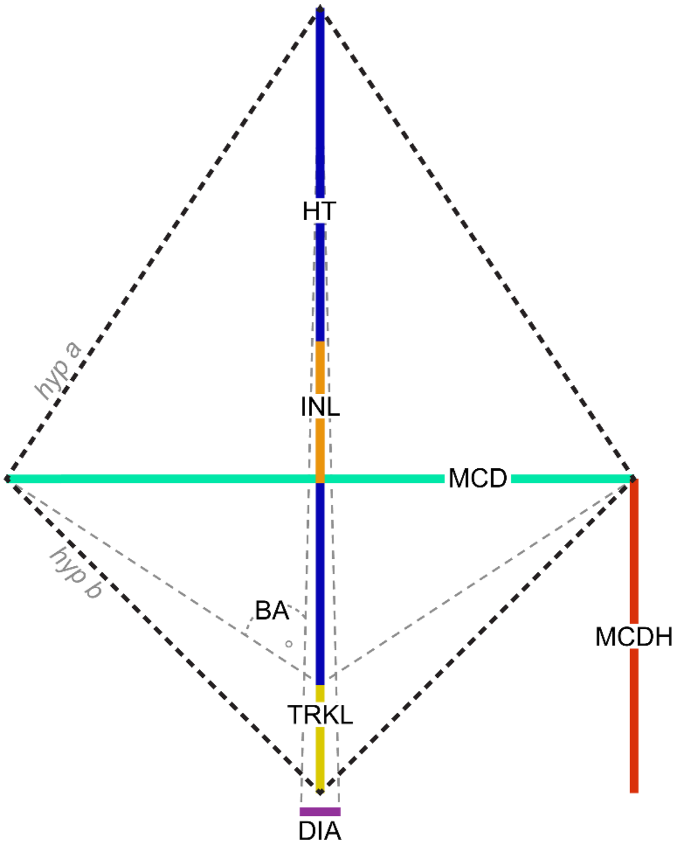

Fig. S4.

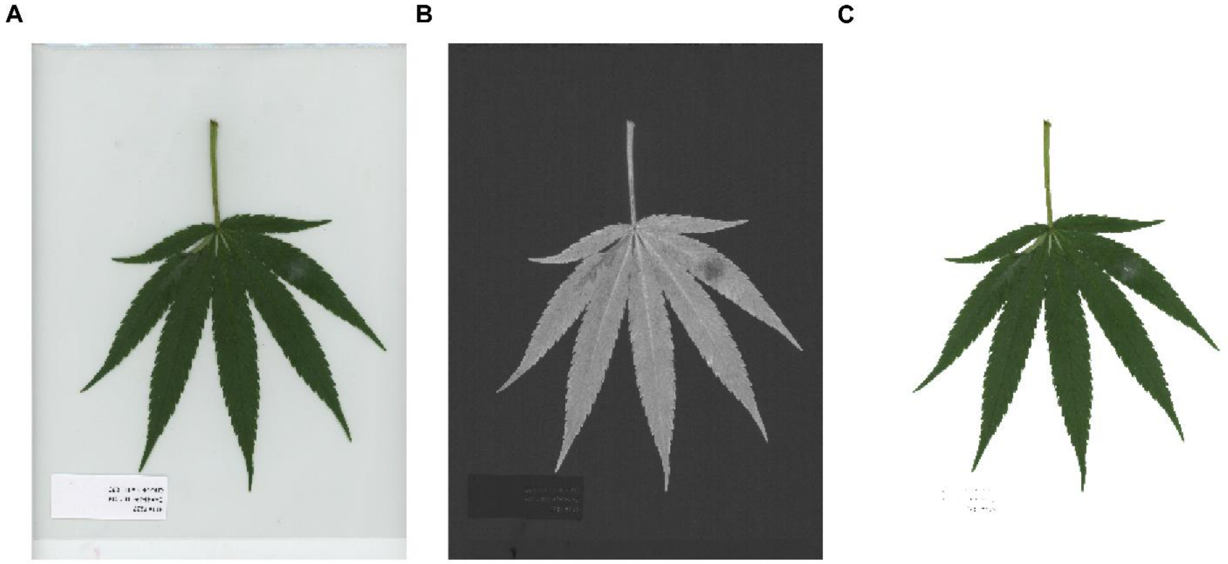

Fig. S5.

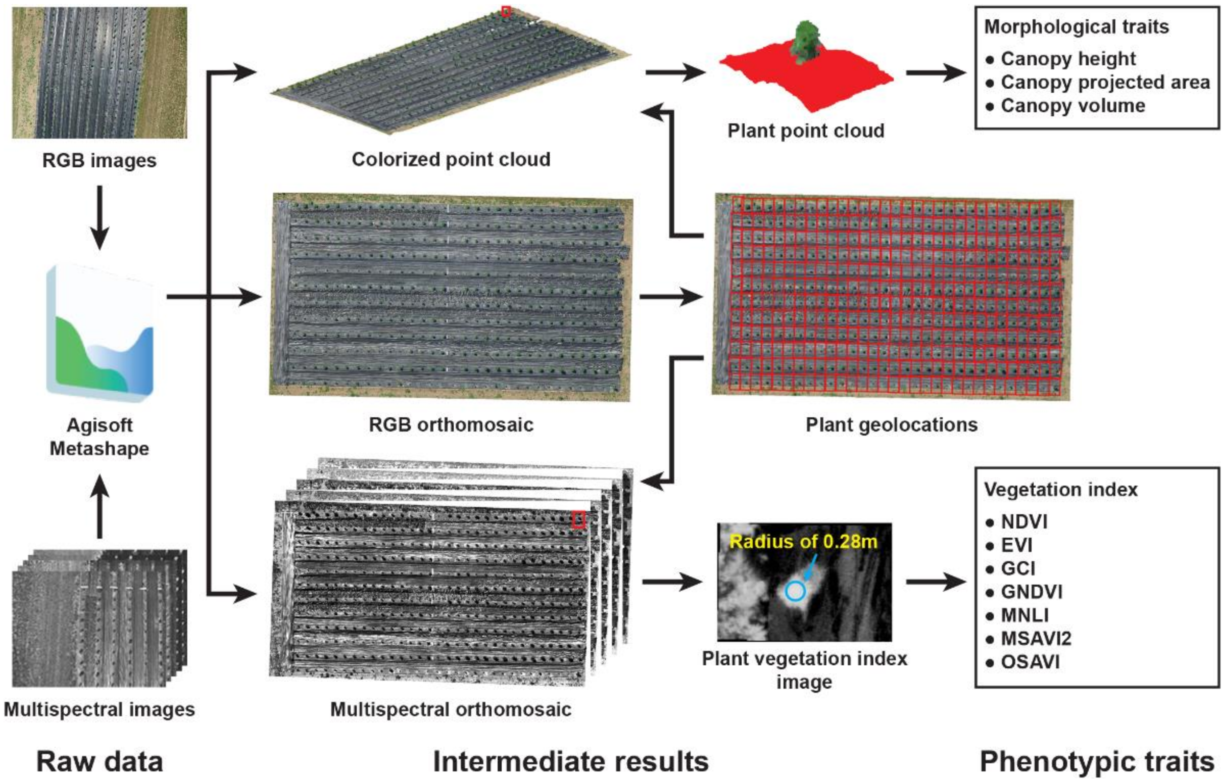
RGB images
Colorized point cloud
Plant point cloud
Morphological traits
• Canopy height
• Canopy projected area
• Canopy volume
Agisoft
Metashape
RGB orthomosaic
Plant geolocations
Vegetation index
• NDVI
• EVI
• GCI
• GNDVI
• MNLI
• MSAVI2
• OSAVI
Radius of 0.28m
Plant vegetation index image
Multispectral images
Multispectral orthomosaic
Raw data
Intermediate results
Phenotypic traits

Fig. S6.

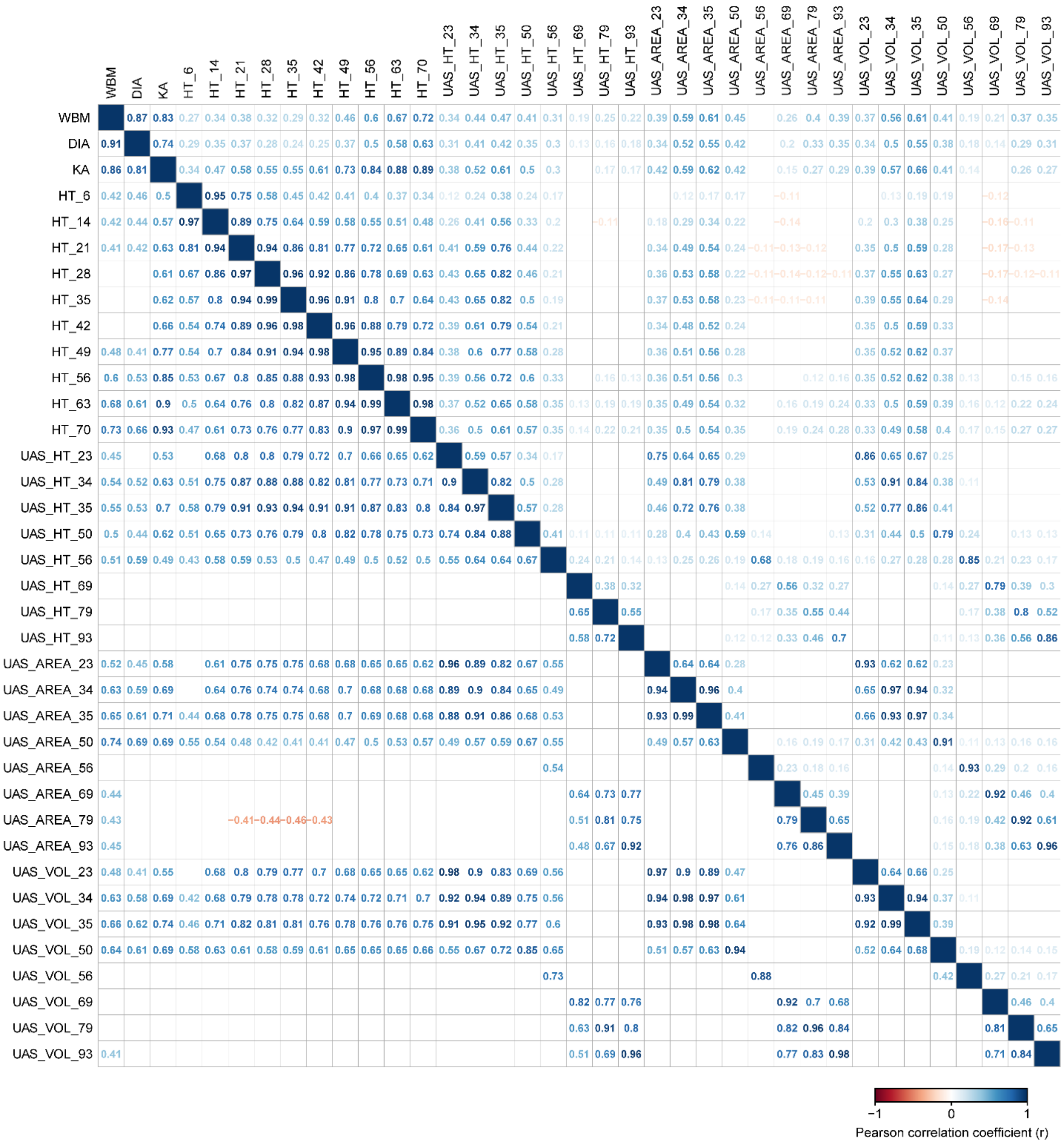
WBM
DIA
KA
HT_6
HT_14
HT_21
HT_28
HT_35
HT_42
HT_49
HT_56
HT_63
HT_70
UAS_HT_23
UAS_HT_34
UAS_HT_35
UAS_HT_50
UAS_HT_56
UAS_HT_69
UAS_HT_79
UAS_HT_93
UAS_AREA_23
UAS_AREA_34
UAS_AREA_35
UAS_AREA_50
UAS_AREA_56
UAS_AREA_69
UAS_AREA_79
UAS_AREA_93
UAS_VOL_23
UAS_VOL_34
UAS_VOL_35
UAS_VOL_50
UAS_VOL_56
UAS_VOL_69
UAS_VOL_79
UAS_VOL_93
−1
0
1
Pearson correlation coefficient (r)

Fig. S7.

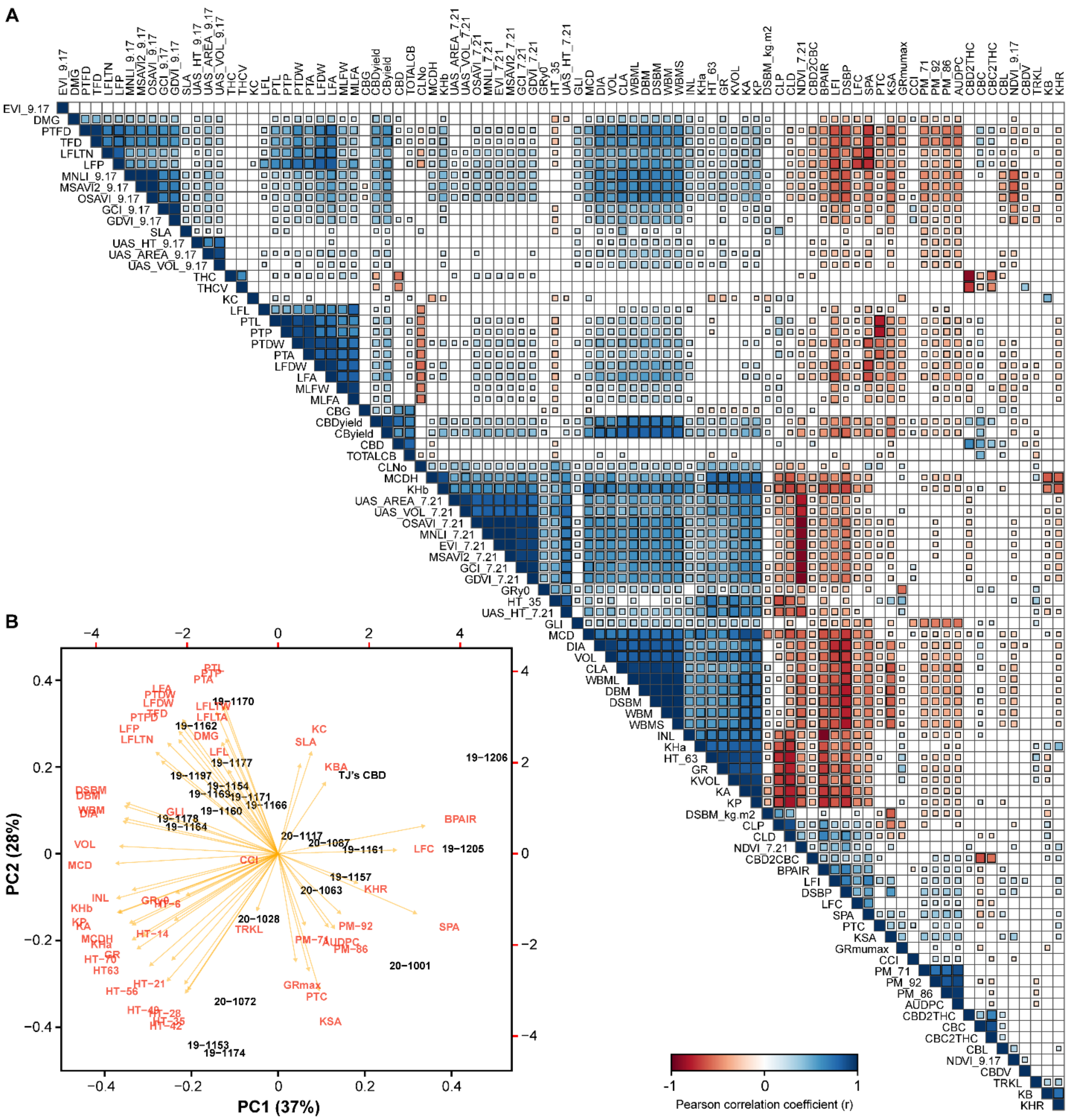

Fig. S8.

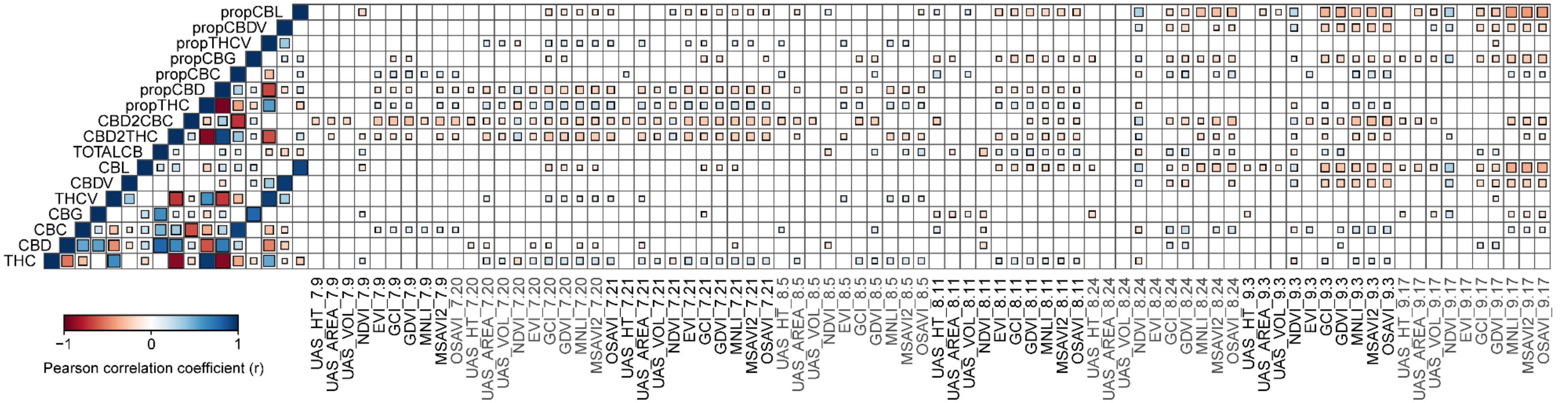
propCBL
propCBDV
propTHCV
propCBG
propCBC
propCBD
propTHC
CBD2CBC
CBD2THC
TOTALCB
CBL
CBDV
THCV
CBG
CBC
CBD
THC
−1
0
1
Pearson correlation coefficient (r)
UAS_HT_7.9
UAS_AREA_7.9
UAS_VOL_7.9
NDVI_7.9
EVI_7.9
GCI_7.9
GDVI_7.9
MNLI_7.9
MSAVI2_7.9
OSAVI_7.20
UAS_HT_7.20
UAS_AREA_7.20
UAS_VOL_7.20
NDVI_7.20
EVI_7.20
GCI_7.20
GDVI_7.20
MNLI_7.20
MSAVI2_7.20
OSAVI_7.21
UAS_HT_7.21
UAS_AREA_7.21
UAS_VOL_7.21
NDVI_7.21
EVI_7.21
GCI_7.21
GDVI_7.21
MNLI_7.21
MSAVI2_7.21
OSAVI_7.21
UAS_HT_8.5
UAS_AREA_8.5
UAS_VOL_8.5
NDVI_8.5
EVI_8.5
GCI_8.5
GDVI_8.5
MNLI_8.5
MSAVI2_8.5
OSAVI_8.5
UAS_HT_8.11
UAS_AREA_8.11
UAS_VOL_8.11
NDVI_8.11
EVI_8.11
GCI_8.11
GDVI_8.11
MNLI_8.11
MSAVI2_8.11
OSAVI_8.11
UAS_HT_8.24
UAS_AREA_8.24
UAS_VOL_8.24
NDVI_8.24
EVI_8.24
GCI_8.24
GDVI_8.24
MNLI_8.24
MSAVI2_8.24
OSAVI_8.24
UAS_HT_9.3
UAS_AREA_9.3
UAS_VOL_9.3
NDVI_9.3
EVI_9.3
GCI_9.3
GDVI_9.3
MNLI_9.3
MSAVI2_9.3
OSAVI_9.3
UAS_HT_9.17
UAS_AREA_9.17
UAS_VOL_9.17
NDVI_9.17
EVI_9.17
GCI_9.17
GDVI_9.17
MNLI_9.17
MSAVI2_9.17
OSAVI_9.17
